# Obesity: a challenge for emergency medical teams—a narrative review

**DOI:** 10.3389/fpubh.2025.1659924

**Published:** 2025-09-29

**Authors:** Michał Czapla, Raúl Juárez-Vela, Aleksander Mickiewicz, Andrzej Raczyński, Krzysztof Griesmann, Kamil Kędzierski, Jakub Wojciechowski, Michał Burzński, Olga Fedorowicz, Mariusz Koral, Damian Kowalczyk, Jacek Smereka

**Affiliations:** ^1^Department of Emergency Medical Service, Faculty of Nursing and Midwifery, Wroclaw Medical University, Wroclaw, Poland; ^2^Group of Research in Care (GRUPAC), Faculty of Health Science, University of La Rioja, Logroño, Spain; ^3^Department of Clinical Pharmacology, Faculty of Pharmacy, Wroclaw Medical University, Wroclaw, Poland; ^4^Medical Simulation Center, Faculty of Medicine, Wroclaw Medical University, Wroclaw, Poland; ^5^Department of Emergency Medical Service, Academy of Medical, Applied and Holistic Sciences, Warszawa, Poland

**Keywords:** obesity, emergency medical services, prehospital care, cardiopulmonary resuscitation, patient transport

## Abstract

The global rise in obesity presents significant challenges for emergency medical services (EMS), particularly in prehospital care settings. This narrative review examines the multifaceted impact of obesity on emergency interventions, focusing on airway management, resuscitation, vascular access, pharmacological considerations, transport logistics, and point-of-care ultrasound. Evidence indicates that excess adipose tissue alters the biomechanics of chest compressions, increases thoracic bioimpedance during defibrillation, and complicates airway management—especially in cases when advanced airway devices are required. Additionally, pharmacokinetic and pharmacodynamic differences in this population necessitate careful dose adjustment based on drug solubility and body composition. Obtaining vascular access in individuals with obesity is frequently hampered by anatomical constraints, often requiring ultrasound-guided cannulation or intraosseous access. Prehospital transport introduces additional logistical and ergonomic challenges, exacerbated by limited availability of appropriately sized equipment, weight-restricted airframes in Helicopter Emergency Medical Service (HEMS) operations, and difficulty in transferring patients from the scene. Meanwhile, ultrasonographic imaging is technically more demanding and often diagnostically limited in this group due to tissue depth and image degradation. These factors collectively compromise both the speed and quality of emergency care. This review highlights the importance of developing tailored protocols, specialized equipment, and targeted training for Emergency Medical System (EMS) providers who manage patients with obesity. Given the increasing prevalence of patients with obesity in the emergency setting, early identification and anticipatory planning are critical for improving patient outcomes. Addressing these operational and clinical challenges must become a priority for modern EMS.

## 1 Introduction

Obesity has reached unprecedented levels worldwide, affecting over 1 billion individuals, including more than 880 million adults and 159 million children as of 2022 (1). It is no longer considered merely a risk factor but a chronic, multifactorial disease that significantly increases morbidity and mortality across a broad spectrum of conditions, particularly cardiovascular disease (CVD). The global burden of obesity is increasing in both high-income and low- and middle-income countries, with an alarming acceleration in severe obesity and related complications.

Obesity is commonly diagnosed using the body mass index (BMI), with a threshold of ≥30 kg/m^2^ meeting criteria for obesity and ≥40 kg/m^2^ defining severe obesity (2, 3). However, BMI alone fails to capture the full complexity of adiposity-related health risks (4). It does not account for fat distribution, visceral adiposity, or body composition and can misclassify individuals with high muscle mass or metabolically healthy profiles (4). Recent evidence highlights the importance of incorporating additional markers such as waist circumference, waist-to-height ratio, and metabolic parameters to refine risk stratification (5). Moreover, the recognition of distinct obesity phenotypes—such as metabolically unhealthy normal weight (MUNW) or metabolically healthy obesity (MHO)—underscores the need for more nuanced approaches to diagnosis and clinical decision-making (5).

For emergency medical teams (EMT), the implications of this epidemic are far-reaching. Obesity interferes with nearly every aspect of acute and prehospital care, impairing effective chest compressions during cardiopulmonary resuscitation, complicating airway management, increasing thoracic bioimpedance during defibrillation, altering the pharmacokinetics and pharmacodynamics of critical medications, and posing significant logistical challenges in patient transport and evacuation (6, 7). Furthermore, Emergency Medical System (EMS) providers often lack access to equipment and protocols optimized for patients with obesity, leading to suboptimal care and increased occupational risk.

While lifestyle modification and nutritional strategies play a central role in preventing and managing obesity in the long such interventions offer limited utility in the high-stakes setting of acute medical emergencies (8, 9). The reality is that EMS teams are increasingly required to deliver life-saving care to patients with obesity in environments that are poorly adapted to their needs (10).

Despite the scale and clinical complexity of the problem, no unified or comprehensive international guidelines currently exist to direct emergency care specifically for patients with obesity. A recent joint position statement from the Brazilian Association of Emergency Medicine (ABRAMEDE) and the Brazilian Association for the Study of Obesity and Metabolic Syndrome (ABESO) has addressed challenges in the emergency department context, but evidence-based, globally applicable recommendations for prehospital and EMS care remain lacking (11). Therefore, this narrative review aims to highlight the most frequent and clinically significant challenges that obesity poses in emergency settings, particularly those encountered by EMS teams during life-threatening situations.

## 2 Materials and methods

A narrative review was conducted to explore the clinical, operational, and pharmacological challenges associated with managing individuals with obesity in prehospital and emergency care settings. Relevant literature was identified through a structured search of MEDLINE (via PubMed), EMBASE, and the Cochrane Central Register of Controlled Trials (CENTRAL). Search terms included combinations of Medical Subject Headings (MeSH) and relevant free-text keywords related to obesity, emergency medicine, and prehospital interventions (e.g., “airway management,” “chest compressions,” “vascular and intraosseous access,” “defibrillation,” “prehospital ultrasound,” “hemorrhage control,” “patient transport,” “Helicopter Emergency Medical Service/HEMS,” “prehospital pharmacotherapy”). The search covered literature published between January 2000 and January 2025, with the final search performed on 15 January 2025. Inclusion criteria comprised peer-reviewed articles, clinical guidelines, case reports, and observational studies in English. Editorials, letters, and non–peer-reviewed materials were generally excluded from the analytic synthesis, although selected editorials were cited in the Introduction for contextual purposes. In cases of multiple guideline versions, the most recent iteration was included.

## 3 Obesity in emergency prehospital care

### 3.1 Chest compressions in patients with obesity

Effective chest compressions remain a critical component of cardiopulmonary resuscitation (CPR); however, their quality is often significantly compromised in individuals with obesity. The currently recommended compression depth of 50–60 mm (12) may not be universally appropriate, particularly in patients with elevated BMI. Studies suggest that increasing thoracic wall thickness in individuals with obesity directly affects the depth, recoil, and overall efficacy of compressions.

Simulation-based data indicate that healthcare providers achieve significantly fewer high-quality compressions on manikins representing obesity and severe (class III) obesity than on standard-size models, with both rescuer BMI and muscular strength influencing outcomes (13). Severe (class III) obesity has been associated with reduced adequacy of thoracic compressions in simulation, with only 29.3% of compressions on severe-obesity manikins meeting American Heart Association (AHA) standards, compared with >80% on models representing normal weight (14). From a biomechanical standpoint, the uniform application of a 50 mm compression depth has been challenged. Computed Tomography (CT)-based analyses suggest that in patients with obesity, such absolute targets may result in excessive residual compression, potentially causing harm. As a result, some researchers recommend tailoring compression depth to external chest dimensions, such as using a fixed percentage of the anterior–posterior diameter rather than an absolute depth (15).

Operator fatigue is another critical factor. Although adequate compression depth may be achieved at the onset, performance tends to deteriorate rapidly over time—particularly in high-resistance simulations reflective of obese thoraxes—underscoring the importance of rotating compressors approximately every 2 min to maintain quality (16). These observations are consistent with the findings of a recent ILCOR scoping review, which highlighted that CPR quality is rarely reported in relation to obesity and that no evidence-based adaptations of resuscitation techniques currently exist in this population (17). In addition to internal biomechanical resistance, external barriers such as excessive subcutaneous tissue may further impair compression depth and chest recoil. These soft tissue layers have been shown to function similarly to protective equipment (e.g., football shoulder pads), disrupting hand placement and reducing the effectiveness of compressions (18). Even relatively pliable obstructions—such as sports gear—can diminish compression quality, although typically to a lesser extent than the bioimpedance caused by high body mass (19).

Taken together, the literature consistently underscores the need for modified CPR protocols in patients with obesity. This includes re-evaluation of recommended compression depths based on thoracic dimensions, emphasis on proper rescuer technique and physical capacity, frequent rotation to reduce fatigue, and consideration of mechanical compression devices to ensure consistent performance. Despite growing evidence that fixed compression depths may not be appropriate for all body types, both the European Resuscitation Council (ERC, 2021) and the most recent international consensus on resuscitation science (ILCOR/AHA/ERC, 2024) continue to recommend a uniform chest compression depth of 5–6 cm in adults, without specific adaptations for individuals with obesity (20). Without such adjustments, CPR quality may be compromised, potentially reducing survival rates and placing excessive strain on emergency responders. While mechanical chest compression devices may be considered, their use in patients with obesity is limited by chest dimensions and device specifications (e.g., chest width, sternum height). Currently, there is a lack of clinical research evaluating their efficacy and safety in this population. Theoretical application remains possible within defined manufacturer limits; for example, the LUCAS 2 device allows use up to a chest height of 305–340 mm and a weight of 136 kg, provided the device can be applied correctly (21). This area remains underexplored and warrants further investigation, particularly in relation to emergency responders’ performance and patient outcomes. As highlighted by Telson et al., one promising area for future research involves the integration of mechanical and feedback compression systems to enhance CPR quality in challenging scenarios (13). However, the current evidence base is heterogeneous and derives largely from manikin studies or imaging simulations rather than clinical outcome data. While some authors argue for individualized, dimension-based compression depths, others caution that such adaptations could complicate training and reduce consistency in real-life resuscitation. There is also conflicting evidence regarding the utility of mechanical compression devices in patients with obesity: although they may reduce rescuer fatigue, their applicability is limited by chest dimensions, and no robust clinical trials have confirmed survival benefits in this subgroup. This highlights a major gap in the literature and underscores the need for prospective studies evaluating CPR strategies specifically in populations with obesity.

### 3.2 Airway management challenges in patients with obesity

Patients with obesity may present substantial challenges in various aspects of airway management, including bag-valve-mask (BVM) ventilation and the use of advanced airway devices. Notably, BVM ventilation with a self-inflating bag and face mask is often significantly more challenging in this population compared to individuals without obesity (22). A meta-analysis by Hung KC et al. identified 13 major risk factors for predicting difficult BVM ventilation. Drawing on data from 20 observational studies involving 335,846 patients, the analysis reported a 6.1% incidence of difficult BVM ventilation in the general population and 14.4% among individuals with obesity. Obesity was among the most significant predictors (23).

Tracheal intubation in patients with obesity remains a critical yet complex intervention, requiring the use of validated predictive scales (24). In emergency medical contexts, especially prehospital settings, the need for intubation often arises abruptly—during sudden clinical deterioration or cardiac arrest. Factors such as increased neck circumference, excessive soft tissue, upper airway collapsibility, urgency of care, and reduced patient cooperation compound the challenge. Obesity, as an easily identifiable physical trait, may serve as a pragmatic indicator of difficult intubation for paramedics operating under time constraints (25, 26).

This view is supported by data from the United States. Hubble MW et al., in a retrospective study using national EMS data covering 45,344 patients, found that higher body weight was negatively associated with intubation success. A weaker yet positive trend was observed for blind insertion of supraglottic airway devices (SADs). The authors concluded that body weight could be an accessible and clinically useful predictor of intubation difficulty (27). More recently prospective study demonstrated that ultrasound-measured skin-to-epiglottis distance is a reliable predictor of difficult intubation in patients with obesity, offering a simple bedside tool to enhance risk stratification (28). Failure to establish a secure airway—whether through intubation or alternative means—can lead to hypoxaemia, with the potential for fatal or irreversible neurological injury (29). Airway difficulties are to be expected in individuals with severe obesity, commonly defined as a BMI exceeding 40 kg/m^2^ (30). However, studies on intubation success among patients with obesity versus those without obesity yield mixed findings. While some report increased failure rates in individuals with obesity subgroup, others find no significant differences (31, 32). Complementing these findings, a 2025 prospective observational study reported that weight loss following bariatric surgery significantly improves airway classification scores and reduces the incidence of difficult intubation, underscoring the dynamic nature of airway risk in this population (33).

In airway management, it is important to distinguish between difficulty visualizing the glottis and difficulty advancing the endotracheal tube—both of which can occur independently. Although videolaryngoscopy and optical-guided tubes improve visualization, advancing the tube may remain technically demanding. Proper positioning and the use of bougies or other introducers may enhance success (34). The “head-elevated laryngoscopy position” (HELP), also known as the “ramp” position—where the upper body is elevated 25–30 degrees to align the sternal notch with the external auditory meatus—has been shown to facilitate mask ventilation and intubation in patients with severe obesity. A study by Lee S. et al. confirmed these benefits, including reduced intubation time (35). To date, no studies have demonstrated the effectiveness of HELP or ramp positioning in prehospital care, where paramedics frequently work in floor-level environments. However, the 2021 ERC guidelines indicate that patients with obesity already positioned on a bed need not be transferred to the floor, thus enabling the use of the ramp position when feasible (36).

Insertion of a laryngeal mask airway (LMA) in patients with severe obesity is generally contraindicated in elective settings. However, in emergency scenarios, especially in prehospital care, such contraindications may be outweighed by clinical necessity. In these cases, correct positioning, device size selection, and insertion technique are crucial (37). In patients with a BMI of 30 kg/m^2^, LMA use may be associated with better postoperative pulmonary function and oxygenation than tracheal intubation in surgical contexts (38). Conversely, in those with BMI > 40 kg/m^2^, the risk of SAD misplacement rises significantly—by more than threefold—potentially leading to inadequate ventilation (39).

According to the 2021 ERC guidelines—a recommendation reaffirmed in the 2024 ILCOR consensus—difficult airway management in patients with obesity should prioritize early videolaryngoscopic intubation by the most experienced team member (12, 20). In case of failure (up to three attempts), supraglottic airway devices should be considered. Increased abdominal girth and cephalad diaphragm displacement may necessitate elevated airway pressures, thereby raising the risk of gastric regurgitation (12, 40). Dual-lumen SADs with high-pressure cuffs and oesophagal vent channels are thus preferred (38). A meta-analysis assessing patient-related predictors of difficult BVM ventilation identified obesity, increased neck circumference, and habitual snoring as significant risk factors (23). In patients with obesity, potential airway difficulties should always be considered as part of prehospital management planning. Despite these insights, the evidence base remains heterogeneous and context-dependent. While videolaryngoscopy is generally advocated in guidelines for patients with obesity, real-world data are inconsistent, with some studies showing no clear advantage over conventional laryngoscopy. Conversely, supraglottic devices may offer rapid rescue ventilation, but their higher risk of malposition in patients with severe obesity raises safety concerns. Importantly, most comparative studies derive from elective surgical settings, and robust prehospital trials are almost entirely lacking. This discrepancy underscores a critical evidence gap: optimal airway strategies for patients with obesity in emergency out-of-hospital environments remain uncertain and require targeted investigation.

### 3.3 Vascular access in patients with obesity

Establishing vascular access in patients with obesity may be particularly challenging due to anatomical and physiological changes associated with excessive body weight. Difficult intravenous access (DIVA) is defined as two or more failed cannulation attempts or the need to employ advanced techniques such as ultrasound or near-infrared devices (41). Studies indicate a high prevalence of vascular access difficulties in the prehospital management of patients with obesity (41, 42). It is estimated that DIVA occurs in 51 to 90% of all patients (42). The primary challenge in this population is the presence of excess adipose tissue, which contributes to anatomical variations, deeper vein location, reduced ability to palpate veins, and lack of visible landmarks (43).

Another common limitation is the inadequacy of standard-length cannulas to reach deeper veins. It is recommended that a cannula occupy no more than one-third of the vein’s internal diameter and extend no more than two-thirds of its length within the vessel (43). Long peripheral catheters (LPCs) and midline catheters (MCs) are frequently used to overcome these limitations. Their use increases first-attempt success rates by up to 20% and is associated with lower complication rates (25–70%) compared to conventional short peripheral catheters (43, 44). However, their insertion requires operator training and aseptic conditions, which may not be feasible in prehospital settings where rapid vascular access is essential. In these scenarios, conventional short IV catheters are most often employed (45). Studies show that the antecubital fossa is the most commonly used site for vascular access in this group. According to research by Vison H., cannulation sites in patients with DIVA include the antecubital fossa (65%) and forearm (14%) (46).

The use of ultrasound guidance under challenging cases significantly facilitates vascular access. Pablo Blanco’s research demonstrated that ultrasound guidance increased vascular access success rates to 90% in patients with DIVA, compared to 25–30% with traditional techniques. The main limitation reported was early catheter failure, with 50% of ultrasound-guided catheters becoming occluded within 24 h (43). Several studies have demonstrated that external jugular vein cannulation, when performed under ultrasound guidance, is a safe and rarely complicated procedure in emergency prehospital care. It is particularly recommended in urgent situations where rapid access is required. However, in patients with severe obesity, anatomical limitations such as short necks and excess cervical adipose tissue may hinder access, making this method less reliable in this subgroup (47–49).

In a study by Keyes et al. involving brachial and basilic vein cannulation in patients with DIVA, including those with obesity, access was achieved under ultrasound guidance using a 2-inch 18–20G catheter. The average cannulation time was 77 s. In 10% of patients, complications included pain during infusion, catheter dislodgement, occlusion, and accidental arterial puncture. The authors concluded that ultrasound-guided access to these veins is quick and effective, suitable for both prehospital and hospital care settings (50). Chinnock conducted a study on ultrasound-guided cannulation in patients with DIVA, reporting a 63% success rate. The basilic and brachial veins were the most common sites, with basilic vein cannulation yielding a higher success rate of 71% (51).

In patients with obesity, 18G or 20G peripheral IV catheters are typically used, especially when veins are problematic to locate or lie deeper under the skin. These sizes allow adequate flow for drug and fluid administration in patients with high adipose tissue content. The 18G catheter, with a 45 mm needle, is often preferred to access deeper veins. In contrast, 22G catheters are used when veins are easily accessible and rapid fluid administration is not required. In prehospital care, selecting between 18G and 20G catheters depends on vein visibility, diameter, and the patient’s condition. Larger-bore catheters such as 16G and 17G, although offering greater flow rates, are not recommended in patients with obesity due to their high failure rates. The literature emphasizes the importance of using ultrasound guidance to improve first-attempt success rates (52).

Intraosseous access is increasingly utilized in prehospital care for patients with DIVA. Its use is particularly justified in individuals with BMI ≥ 40.0 kg/m (2) when intravenous access is not achievable. Studies from the United States show that intraosseous access accounts for only 0.4% of all prehospital procedures, typically following at least one failed IV attempt. Although data on obesity-specific use are limited, intraosseous access had a first-attempt success rate of 87%, compared to 72% for IV access. Overall success rates were 100% for intraosseous versus 75% for IV access. Conventional IV catheters are more prone to complications such as occlusion or dislodgement. In contrast, intraosseous lines are more stable and do not require adjunctive tools like ultrasound (53). The proximal tibia is the most commonly selected site for intraosseous access, used in 75% of cases according to Torres F. The procedure is associated with a 99% success rate and is considered fast and safe even in patients with extreme obesity (54). According to Kehrl et al., in adults with obesity and a palpable tibial tuberosity or BMI ≤ 43 kg/m^2^, a 25 mm intraosseous needle is sufficient for both proximal and distal tibial access. For access via the proximal humerus in patients with obesity, a 45 mm intraosseous needle is recommended (55). Current literature highlights a lack of research specifically addressing vascular access in patients with obesity within prehospital settings. Most studies focus on DIVA generally without clearly delineating obesity as a subgroup. Further targeted studies are needed to bridge this gap.

### 3.4 Defibrillation

Thoracic bioimpedance is a key factor in the effectiveness of defibrillation, as it determines the resistance the electrical current must overcome to reach the heart. In individuals with a BMI over 30, the presence of excess adipose and soft tissue may increase bioimpedance, potentially reducing current flow and the likelihood of successful rhythm restoration (56). Reported trans-thoracic impedance in adults typically ranges from 51 to 112 ohms, with a mean of around 76.7 ohms. This variability can result from multiple factors, including electrode size and placement, chest dimensions, lung volume, hemoglobin level, and individual anatomical and physiological differences (56, 57). Standard defibrillation protocols recommend initial energy settings of 150–200 J (12). However, due to elevated impedance in this group, higher energy delivery may be warranted to improve the likelihood of rhythm restoration. In the randomized BEST-AF trial, Glover et al. demonstrated that a single fixed 200 J shock was more effective in restoring sinus rhythm during cardioversion—particularly in patients with a BMI > 25 kg/m^2^—compared to an escalating energy protocol, highlighting the potential benefit of higher initial energy settings in individuals with overweight or obesity (58).

The ERC 2021 guidelines recommend that the first shock be administered according to the manufacturer’s instructions, with subsequent shocks at maximum available energy if the initial attempt is unsuccessful. This approach is reaffirmed in the 2023 AHA guidelines and the 2024 ILCOR consensus, which do not provide obesity-specific modifications (12, 20, 59). Electrode placement and interface quality are also critical. In individuals with obesity, excessive subcutaneous fat can compromise effective current delivery in the standard anterior-lateral configuration. A randomized controlled trial assessing cardioversion of atrial fibrillation in this group demonstrated reduced success with adhesive pads and standard energy levels, whereas manual paddles or applied pressure significantly improved outcomes by enhancing contact and reducing impedance (60). Alternative approaches, such as anterior–posterior positioning, may also improve current flow across the myocardium. Evidence suggests that this configuration may outperform the anterior–lateral placement in individuals with elevated BMI. Manual paddles, particularly when pressure is applied, offer another advantage when adhesive pads fail to provide sufficient skin contact (61).

Double sequential defibrillation (DSD), which involves two defibrillators delivering shocks either simultaneously or within milliseconds, has been explored as a solution in refractory ventricular fibrillation. While evidence remains limited, early reports suggest it may be beneficial in individuals with elevated BMI, particularly when standard high-energy defibrillation fails (62). However, a recent meta-analysis did not show significant superiority of DSD over conventional methods in terms of ROSC or survival rates (63). A recent RCT by Aymond et al. showed that dual cardioversion was more effective than single-shock therapy in patients with obesity and atrial fibrillation, with 98% versus 86% success, respectively. Those who failed single cardioversion responded to dual therapy, suggesting that this strategy may also prove beneficial in cases of VF/VT arrest in this population (64). Current ERC guidelines state that there is insufficient evidence to recommend the routine use of DSD for cases of refractory ventricular fibrillation. Despite growing interest and some promising results in select populations, including individuals with obesity, the lack of robust, high-quality evidence prevents DSD from being incorporated into standard resuscitation protocols at this time (12). Nevertheless, the current evidence should be interpreted with caution. Most studies investigating vascular access difficulties do not specifically stratify outcomes for patients with obesity, limiting the ability to draw firm conclusions for this population. Reports on ultrasound-guided cannulation show variable success and high early failure rates, raising questions about generalizability to prehospital settings where time and equipment are constrained. Similarly, while intraosseous access demonstrates high success rates, its low utilization in routine EMS practice suggests a significant gap between evidence and implementation. These inconsistencies highlight the need for targeted prospective research to establish best practices for vascular access in patients with obesity in prehospital care.

### 3.5 Management of hemorrhage in patients with obesity

Massive hemorrhage remains one of the leading preventable causes of death following trauma. To reduce mortality and minimize the time to effective hemorrhage control, the CARE approach should be implemented. This structured protocol prioritizes C—bleeding control—before progressing to the standard patient evaluation sequence: A for airway assessment, R for respiratory evaluation, and E for exposure and prevention of hypothermia (65, 66, 119). In cases of life-threatening bleeding, three anatomical zones require specific management strategies. Extremity hemorrhages, including those from both upper and lower limbs, are managed by applying tourniquets. Junctional areas such as the neck, axillae, and groin require direct pressure or wound packing using hemostatic dressings or rolled gauze. For injuries to the thoracic or abdominal regions, sealing the wound with occlusive or vented dressings is indicated, while packing these sites is categorically contraindicated (67–69). Additionally, pelvic trauma or suspected pelvic injury must be addressed with appropriate stabilization measures. In patients with obesity, each of these zones presents unique technical challenges.

For extremity hemorrhages, tourniquet application follows one of two approaches. When the bleeding site is visible, the tourniquet should be applied 5–7 cm proximal to the wound, avoiding joints. If the bleeding source is not visible, the tourniquet is applied as proximally as possible (70). In all cases, the device must be tightened until hemorrhage ceases, and the time of application must be documented. In first aid settings, the tourniquet should remain in place until emergency personnel arrive. However, in prehospital emergency medical interventions, the provider may consider converting the tourniquet to a pressure dressing if appropriate (71, 72). One of the most significant limitations in patients with obesity is the circumference of the limb, which may exceed the capacity of standard tourniquets. For reference, the Black Front tourniquet accommodates limb circumferences up to 80 cm, the widely used CAT Gen.7 up to 82 cm (73), and the SOF Gen.5 up to 101.6 cm—the longest commercially available option (74). While the application procedure remains the same across body types, a tourniquet that is too short may fail to compress the vessel effectively.

Additionally, excessive soft tissue mass, both muscular and adipose, can hinder effective hemorrhage control (71). Management of junctional bleeding typically requires wound packing. Ideally, a hemostatic dressing should be used, suitable both for first aid and professional emergency medical care. In individuals with obesity, the challenges include locating the bleeding source and the need for a larger volume of packing material due to increased tissue depth. Adipose tissue may complicate both access and effective tamponade. Once the wound is packed, firm direct pressure should be applied, and a compression dressing—preferably an Israeli-style bandage—should be used to secure it (75).

For injuries involving the thorax or abdomen, the recommended approach is sealing the wound with occlusive or vented dressings. In cases of junctional or internal torso bleeding, specialized devices designed to compress major vessels may be employed, such as the SAM Junctional Tourniquet (maximum circumference: 111 cm) or the AAJT-S (up to 156 cm) (70). Proper device selection and use must be tailored to the patient’s body habitus, specifically the circumference at the intended application site. Pelvic fractures or suspected pelvic trauma should be managed using dedicated pelvic binders. Devices such as the T-POD (suitable for patients over 22.5 kg, with a maximum pelvic circumference of 160 cm) or the SAM Pelvic Sling II (available in multiple sizes, with a maximum circumference of 137.16 cm) are commonly used (70, 76). As with other hemorrhage control devices, patient girth is a key factor in determining both applicability and efficacy. In summary, the body habitus of patients with obesity presents significant challenges in the control of massive hemorrhage. The circumference of various body regions may preclude the effective use of dedicated hemorrhage control equipment, potentially impacting survival in this vulnerable patient group (77). Despite the availability of multiple hemorrhage control devices, evidence regarding their effectiveness in patients with obesity is limited. Most data are derived from general trauma populations or device specifications rather than trials explicitly including individuals with larger body habitus. This creates uncertainty about real-world performance when limb or pelvic circumferences exceed manufacturer-recommended limits. Furthermore, no standardized protocols currently exist for prehospital ‘bariatric kits’ to address such challenges. These gaps highlight the need for targeted studies evaluating hemorrhage control strategies in patients with obesity and for system-level planning to ensure appropriate equipment availability.

### 3.6 Pharmacokinetic and pharmacodynamic in individuals with obesity

Physiological and anthropometric changes observed in individuals with obesity significantly alter the pharmacokinetic (PK) and pharmacodynamic (PD) profiles of many drugs, potentially impacting the effectiveness of emergency interventions. This presents a substantial clinical challenge, particularly in prehospital care, as existing dosing protocols are often based on clinical trials that rarely include participants with obesity and fail to reflect their unique pharmacokinetic variability (78).

Pharmacokinetic alterations in this population may result in subtherapeutic drug levels or heightened toxicity. Among PK processes, absorption is typically the least affected (79, 80). Emergency medications are generally administered intravenously, bypassing absorption issues; however, in specific circumstances, intramuscular or subcutaneous administration may be necessary and problematic due to the presence of excess adipose tissue—this is particularly relevant for medications like epinephrine used in anaphylaxis. Major PK changes occur during the distribution and elimination phases (81).

Factors including body composition, tissue perfusion, cardiac output, plasma protein binding, membrane transport, acid–base balance, and drug-specific characteristics (82) influence drug distribution. In individuals with obesity, the volume of distribution (Vd) of lipophilic drugs is typically increased, which directly influences the selection of loading doses. Elimination—via hepatic metabolism and renal excretion—may also be impaired. Hepatic steatosis, common in this population, can reduce metabolic clearance and elevate toxicity risk. Opioids, for example, may persist in systemic circulation for longer periods, increasing the likelihood of respiratory depression. The biological half-life (t½) of highly lipophilic agents—such as diazepam, midazolam, propranolol, lidocaine, and verapamil—is frequently prolonged (81). In advanced stages of obesity, impaired renal function can further delay drug clearance and contribute to accumulation.

Pharmacodynamic responses—governing how the body reacts to drugs—are also altered, due to changes in receptor density, tissue sensitivity, and broader systemic dysfunction. Dose adjustments should be tailored to the physicochemical properties of the drug (e.g., lipophilicity) and to anticipated PK/PD alterations resulting from both obesity and the acute clinical scenario (83). Clinically relevant metrics used to guide dose modification include total body weight (TBW), ideal body weight (IBW), adjusted body weight (ABW), and lean body weight (LBW) (84). Loading and maintenance doses may be determined using different references depending on the drug. For example, IBW-based dosing is recommended for benzodiazepines, digoxin, β-blockers, corticosteroids, and aminophylline. LBW is preferred for loading doses of opioids and analgesics including morphine, remifentanil, fentanyl, sufentanil, and paracetamol. TBW may be suitable for initial dosing of verapamil and ketamine, while subsequent doses should revert to IBW calculations (85).

Epinephrine, administered during all types of cardiac arrest, may have reduced efficacy in individuals with obesity. Wang et al. reported that those weighing over 82.5 kg may receive subtherapeutic doses when following standard protocols (86). Higher epinephrine doses (>1 mg) have been associated with increased rates of return of spontaneous circulation (ROSC) and short-term survival, but may be linked to worse neurological outcomes post-resuscitation (87). ERC 2021 guidelines do not support dose escalation during cardiac arrest, a position also maintained in the 2023 AHA guidelines and the 2024 ILCOR consensus, both of which confirm that routine dose escalation is not recommended in patients with obesity (12, 20, 59). Radosevich et al. recommend that vasoactive drugs should be titrated to clinical effect rather than by body weight (88). Emerging technologies may provide novel opportunities to operationalize this recommendation in the prehospital environment. In particular, non-invasive electrical cardiometry (EC) enables real-time measurement of stroke volume, cardiac output, and systemic vascular resistance through thoracic bioimpedance. Feasibility studies in helicopter emergency medical services have demonstrated that EC monitoring can be rapidly deployed without prolonging on-scene time, with acceptable signal quality achieved in the vast majority of patients (89). In critical care settings, EC has shown reasonable correlation with echocardiography, supporting its potential as a continuous hemodynamic monitoring tool (90). Nevertheless, comparisons with transpulmonary thermodilution indicate substantial variability and clinically unacceptable error margins, suggesting that EC cannot yet replace invasive reference methods, particularly in high-risk populations (91). Despite these limitations, EC remains a promising adjunct that may, in the future, enable emergency medical providers to titrate vasoactive drugs according to real-time hemodynamic responses rather than weight-based estimations. Similarly, Taylor et al. found that using ABW to guide initial fluid resuscitation resulted in more favorable outcomes than using TBW or IBW in a large cohort of patients with suspected sepsis (92).

Taken together, PK/PD changes in individuals with obesity necessitate careful consideration in dosing decisions, as suboptimal exposure can compromise therapeutic outcomes or amplify toxicity. These complexities underscore the urgent need for further targeted research on dosing in this growing patient population (80). Importantly, recommendations for dosing adjustments often derive from small cohorts or extrapolations from surgical or intensive care populations, and results are not always consistent across studies. This heterogeneity limits the ability to establish universally applicable protocols for prehospital emergency care.

### 3.7 Point-of-care ultrasound in patients with obesity

Ultrasonography, employed in emergency medicine as a POCUS (Point-of-Care Ultrasound) tool, is a relatively recent yet rapidly evolving method of patient evaluation, often enabling swift and accurate diagnosis. It has proven particularly valuable in the assessment of patients presenting with undifferentiated shock or blunt abdominal trauma (93). Additionally, it plays an important role in the differential diagnosis of dyspnea in prehospital settings, supporting frontline medical personnel in managing acute pulmonary conditions (94). Given the epidemiological patterns observed in individuals with obesity, point-of-care ultrasound may also help diagnose acute cholecystitis, pulmonary embolism, or dissecting abdominal aortic aneurysm (95). Available data on the prehospital use of ultrasound in patients with obesity remain limited—and arguably insufficient. Guillem Cuatrecasas et al. emphasized the relevance of abdominal ultrasound for screening purposes in this group (96).

Cardiopulmonary ultrasound assessments in patients with obesity—particularly for evaluating cardiac function or pulmonary pathology—are often hampered by increased thoracic wall thickness. Technical adaptations such as tissue harmonic imaging, Doppler modes, or contrast-enhanced ultrasound (CEUS) may improve diagnostic yield, but these features are often unavailable in prehospital settings. Despite such challenges, portable ultrasound remains a highly valuable tool for guiding critical decisions, especially in differentiating cardiac from respiratory etiologies of acute symptoms (97–101). Sascha Heinitz et al. have demonstrated that ultrasound examinations in patients with obesity are technically more difficult, with even trained clinicians showing decreased diagnostic performance compared to examinations in individuals without obesity (100). Consequently, one key factor in improving diagnostic accuracy in this population is the appropriate selection of ultrasound probes. Lower-frequency probes (e.g., convex or sector types) offer better penetration—up to 30 cm depth—compared to higher-frequency linear probes. While standard ultrasound devices are typically equipped with 2–5 MHz probes, patients with obesity may benefit from lower-frequency transducers (2–3 MHz), which trade off image resolution for penetration depth. Focused image optimization, such as adjusting scanning depth and focal zones, may be especially important when evaluating parenchymal organs that lie farther from the skin surface (102). Even with such optimizations, achieving good-quality imaging in patients with obesity may require adjusting the probe angle, using maximum scan depth, and applying subcutaneous tissue compression (100). These efforts may still fall short in delivering interpretable images, posing a real challenge in emergency situations.

Obesity is a well-recognized factor that reduces the sensitivity of abdominal ultrasonography performed as part of the FAST (Focused Assessment with Sonography for Trauma) protocol. Studies have shown that in patients with BMI > 30 kg/m^2^, the sensitivity of FAST for detecting intraperitoneal free fluid is approximately 20–25% lower compared to individuals without obesity (103). Similar findings have been reported for the eFAST (Extended FAST) protocol used in emergency departments (104). Rapid diagnosis of blunt abdominal trauma is critical for timely intervention. While FAST remains a cornerstone diagnostic tool in such scenarios, obesity significantly impairs its diagnostic accuracy (103).

Training of prehospital personnel is essential. Ultrasound instruction based solely on theory or simulations may mislead learners into believing the technique is straightforward. To ensure realistic skill acquisition, POCUS training should incorporate patients with obesity and multimorbidity to expose providers to real-world difficulties they may encounter during emergency assessments (105). Contemporary portable ultrasound devices are compact and lightweight, making them suitable for use in across various settings, including public spaces and patients’ homes. Given the technical limitations in patients with obesity, telemedicine capabilities—such as remote consultation with an experienced ultrasonographer—may further enhance diagnostic reliability in this population.

Obesity poses a significant challenge to ultrasound-based diagnosis in emergency care (106). Increased subcutaneous adipose tissue can degrade image quality, making it more difficult to visualize sonographic landmarks and internal organs. Although techniques like tissue harmonic imaging may mitigate some of these effects, examinations remain technically more demanding in patients with obesity. Therefore, it is crucial to adapt scanning techniques through tissue compression, probe angulation, proper transducer selection, and careful adjustment of scan depth and focus. Despite the inherent limitations, ultrasound remains a highly valuable modality in prehospital care, capable of supporting differential diagnosis in patients with obesity during emergencies—provided its limitations are understood and addressed (107). Importantly, most evidence regarding POCUS in patients with obesity derives from in-hospital studies, whereas high-quality prehospital data remain scarce. While adaptations such as probe selection, tissue compression, and harmonic imaging may improve feasibility, these strategies are inconsistently available in frontline EMS equipment. The markedly reduced sensitivity of FAST in this population raises concerns about over-reliance on this modality for trauma triage, yet no validated alternatives exist. This discrepancy highlights both a clinical dilemma and a research gap: although POCUS holds clear potential in emergency care for patients with obesity, its reliability and impact on outcomes in prehospital practice remain insufficiently defined.

### 3.8 Transport considerations for individuals with obesity in EMS and HEMS operations

Medical transport in the prehospital setting involves numerous logistical and organizational challenges that directly affect both patient safety and the effectiveness of emergency medical services (EMS) interventions. A critical issue remains the availability and customization of transport equipment suited for individuals requiring special care, as inadequate ambulance equipment has been associated with prolonged intervention times and increased risk of musculoskeletal injury among EMS personnel (10, 108). Another important factor is the load capacity of ambulance stretchers. Modern powered systems, such as the Stryker Power-PRO XT, are rated up to approximately 318 kg (700 lb) (109) ensuring adequate safety margins for most patients. However, older or manual stretcher models may have substantially lower limits (e.g., 150–250 kg), which can restrict safe transport of patients with severe obesity. This variability highlights the importance of considering equipment specifications when outfitting ambulances and planning bariatric transport. In clinical practice, EMS teams frequently encounter challenges related to the safe transfer of patients, particularly in scenarios that require specialized stretchers or hoisting systems. Transferring individuals with increased body mass is particularly demanding and is associated with elevated rates of musculoskeletal injuries among rescuers. Additionally, the use of non-standard equipment and the delays associated with logistical coordination can prolong the time required to reach definitive care, which is critical in emergencies such as acute coronary syndromes or strokes (10, 110). These concerns underscore the importance of regular training in the deployment of equipment designed for complex transportation scenarios, which leads to reduced error rates and enhanced operational safety. Recent data also suggest that patients with obesity are at higher risk of prolonged on-scene times and hypoxemia during EMS operations, further underscoring the logistical challenges in this population (111).

Despite its increasing relevance, the role of HEMS in this context remains insufficiently documented. Direct-response flights to incident sites present multiple constraints when transporting individuals with severe obesity. Variability in helicopter types, cabin configurations, and medical equipment across HEMS operators globally results in substantial operational heterogeneity when managing patients with increased body habitus (112–115). Technical limitations affecting HEMS operations include aircraft performance (e.g., maximum takeoff weight), stretcher specifications (weight capacity, width, strap length), and external environmental factors such as ambient temperature, barometric pressure, and altitude above sea level—all of which impact the maximum allowable onboard weight (113–117). Additionally, the dimensions of loading doors and available cabin volume may restrict onboard access to the patient, which in turn may compromise safety during transport or in emergency situations requiring evacuation. The landing zone is another critical factor influencing HEMS feasibility. Although air ambulances can typically land in improvised environments requiring minimal space, final authority rests with the helicopter pilot, who must assess obstacles and departure constraints. Landing sites may be located at considerable distances from the incident scene, requiring extended overland transfer using supplementary resources, particularly burdensome for patients with increased body mass (113–115, 118).

To mitigate these challenges, protocols should integrate early estimation of patient weight during dispatch intake—already a standard practice in interfacility transfers—to inform resource allocation and anticipate operational limitations. This allows for the concurrent deployment of additional personnel, ensures timely care for patients exceeding HEMS load capacities, and supports prehospital initiation of advanced interventions while awaiting ground transport. Awareness that the call involves a patient with increased body mass enables better selection of a landing zone during HEMS flight planning. Notably, the current evidence on EMS and HEMS transport of patients with obesity is fragmented and largely descriptive, with little systematic evaluation of outcomes or safety. Considerable variability exists between ambulance services and helicopter operators in terms of equipment availability and load capacity, leading to unequal access to time-critical interventions. This heterogeneity raises concerns about potential disparities in care delivery and underscores the need for standardized bariatric transport protocols and dedicated outcome studies.

Notably, the challenges encountered in resuscitation, airway management, vascular access, pharmacotherapy, and transport are often interconnected and cumulative rather than isolated. To provide a practical overview, Table 1 summarizes strategies to consider. These are derived from available literature and expert opinion; however, the strength of evidence varies and should therefore be interpreted with caution.

**Table 1:** Key challenges in the emergency management of patients with obesity and strategies to consider for potential mitigation.

Domain	Key challenges	Strategies to consider
Cardiopulmonary resuscitation	Reduced chest compression depth and recoil; rescuer fatigue; uncertain benefit of mechanical devices	Frequent compressor rotation; feedback devices; tailored training; evaluation of device limits
Airway management	Difficult bag-mask ventilation; increased risk of failed intubation; SAD malposition in severe obesity	Early videolaryngoscopy; ramped positioning; use of bougies; careful SAD selection; ultrasound predictors (skin-to-epiglottis distance)
Vascular access	Deeper veins, poor visibility/palpation; higher rate of failed IV access	Ultrasound-guided cannulation; use of longer catheters; intraosseous access in refractory cases
Defibrillation	Higher transthoracic impedance; reduced efficacy of standard pad placement	Early use of higher energy; alternative pad positions (anterior–posterior); manual paddles with pressure
Pharmacology	Altered PK/PD; risk of under- or overdosing; limited evidence for weight-based adjustments	Dosing guided by IBW/LBW/ABW depending on drug; titration to effect; novel monitoring tools (e.g., electrical cardiometry)
Transport logistics (EMS/HEMS)	Limited stretcher capacity; rescuer musculoskeletal injuries; restricted helicopter load/cabin space	Bariatric equipment; pre-dispatch weight estimation; specialized training; standardized transport protocols

ABW, adjusted body weight; EMS, emergency medical services; HEMS, helicopter emergency medical services; IBW, ideal body weight; IV, intravenous; LBW, lean body weight; PD, pharmacodynamics; PK, pharmacokinetics; SAD, supraglottic airway device.

## 4 Conclusion

The increasing prevalence of obesity represents a critical and multifaceted challenge for emergency medical systems. Individuals with obesity frequently present with anatomical, physiological, and pharmacological differences that disrupt the assumptions underlying conventional prehospital protocols. These divergences affect virtually every aspect of care—from resuscitation effectiveness and airway management to vascular access, pharmacotherapy, and both ground and air transport logistics. Despite growing awareness, current clinical guidelines remain inadequately adapted to account for these variables, potentially compromising the safety and outcomes of emergency interventions in this high-risk group. There is a pressing need to bridge the gap between existing emergency care standards and the specific demands of patients with obesity. This includes adapting pharmacological dosing schemes, redesigning equipment and training programs, and incorporating body habitus considerations into dispatch, triage, and procedural planning. Failing to recognize obesity as a key modifier of emergency care may not only undermine clinical effectiveness but also perpetuate disparities in access to life-saving interventions. Optimizing prehospital and in-hospital emergency responses for individuals with obesity must, therefore, be recognized as both a clinical and public health priority. Addressing these limitations requires coordinated research efforts, evidence-informed guidelines, and an operational shift toward inclusive, adaptable models of care that reflect the realities of today’s patient populations.
